# Suicide Attempts and Suicides as a Result of Poisoning and under the Influence of Xenobiotics in Poland in 1999–2020

**DOI:** 10.3390/ijerph19042343

**Published:** 2022-02-18

**Authors:** Anna Staniszewska, Dorota Lasota, Aleksandra Kielan, Anna Brytek-Matera

**Affiliations:** 1 Department of Experimental and Clinical Pharmacology, Medical University of Warsaw, 02-097 Warsaw, Poland; dorota.lasota@wum.edu.pl; 2 Department of Public Health, Medical University of Warsaw, 02-097 Warsaw, Poland; aleksandra.kielan@wum.edu.pl; 3 Institute of Psychology, University of Wroclaw, 50-527 Wroclaw, Poland; anna.brytek-matera@uwr.edu.pl

**Keywords:** suicide attempt, epidemiology of suicide, intoxication, Poland

## Abstract

Background: The most common method of suicide in Poland is hanging, especially among men. However, women tend to overdose on medications to commit suicide. Considering suicide attempts, poisoning, which involves overdosing various substances, is the most commonly used method. The purpose of the present study was to analyze suicide attempts by intoxication, suicides, and substances that influenced the state of consciousness of suicide victims in Poland in the years covered by the study. Methods: A descriptive analysis was made based on the data obtained from the registers of the General Police Headquarters of Poland and the Statistics Poland for the years 1999–2020. Results: During the 21-year study period, 161,655 cases of suicide attempts were recorded in Poland, 106,169 of which resulted in suicides. Results showed that out of 14,660 self-poisoning suicide attempts, there were 2258 cases of suicide poisoning deaths in the analyzed study period. According to the data of the General Police Headquarters of Poland, the total number of suicides of all causes was 106,169. Self-poisoning suicides accounted for 2.1% of all cases of suicides. Conclusion: Due to the distribution of suicide registration systems in Poland, data available in this area should be interpreted with caution.

## 1. Introduction

Suicide is a global phenomenon that results from a complex interaction between risk factors and triggers, including alcohol and drugs [1]. Consciousness disorders caused by alcohol consumption or the use of other psychoactive substances are observed in 25%–50% of all suicide victims [2].

According to the World Health Organization (WHO) and the Food and Agriculture Organization of the United Nations (FAO), the most common methods of suicide all over the world are pesticide poisoning in low- and middle-income countries, hanging, which accounts for 50% of suicides in highly developed countries, and jumping from a height, mainly in highly urbanized areas such as China, Hong Kong SAR, and Singapore [3]. Suicidal behavior (suicide method in particular) varies across countries. A high percentage of suicides with firearms (46% of all suicides) is recorded in the highly developed America, whereas the “epidemic” of using barbecue charcoal for suicidal purposes started in Hong Kong in 1998 and quickly spread to Taiwan and China, thus recently becoming the most popular method of suicide in that part of the world. In other countries, hydrogen sulfide and helium are used for suicide purposes (e.g., Japan) [4].

It is important to distinguish between suicide and suicide attempt. Suicide is the act of deliberate taking one’s own life, whereas a suicide attempt is any non-fatal suicide behavior that might have been displayed with or without an intent to take one’s own life [5].

The most common method of suicide in Poland is hanging, especially among men. Women tend to overdose on medications to commit suicide [6,7,8,9,10,11]. Self-poisoning suicide attempts are not uncommon in medical practice; in fact, self-poisoning is one of the most frequently used methods for suicide attempts. Typically, it involves an intentional overdose of various substances. First and foremost, these are benzodiazepine sedatives and hypnotics, antiepileptic drugs (carbamazepine, valproic acid), antidepressants (tricyclic, serotonin reuptake inhibitors), and neuroleptics (classic and atypical). Drug poisoning concerns mostly psychiatric patients and people addicted to drugs and alcohol (they resort to psychotropic drugs when withdrawal symptoms are intensified, or symptoms of acute alcohol intoxication appear) [12]. Other psychoactive substances that are used for suicidal purposes are many over the counter (OTC) drugs, which contain substances that can affect consciousness, behavior, and feeling when consumed in large amounts. The most popular substances of this type are dextromethorphan (a synthetic analog of codeine), ephedrine, and pseudoephedrine [13]. Another group of psychoactive substances that have been present in the statistics of suicides and suicide attempts kept by the Polish police since 2017 are “legal highs”, which contain psychoactive compounds such as N-benzylpiperazine (a substitute for amphetamine), synthetic cannabinoids, and cathinone derivatives (mephedrone, naphyrone) [14].

Drug poisoning can also be of a mixed nature, especially if multi-drug poisoning or alcohol and drug poisoning occurs [12]. Mixing alcohol with drugs intensifies its effects, which may have tragic consequences, especially for people already burdened with mental problems.

The purpose of the present study was to analyze suicide attempts by intoxication, suicides as such, and substances influencing the state of consciousness of suicide victims in Poland in the years covered by the study.

To properly plan preventive actions, it is necessary to determine the scale of a particular problem. Poland does not have one statistical database with the number of suicides. The collection of data by two separate institutions causes a problem with interpreting these data. The aim of this publication was to juxtapose the data from two sources to compare the discrepancies between them. To date, there have been few articles addressing suicide poisoning, and not a single paper has been written that analyzes suicide poisoning statistics over the past 21 years with two data sources. Additionally, in the case of suicide poisoning, knowing what substances are used in suicide attempts may be helpful in limiting access to these substances.

## 2. Materials and Methods

There is no national suicide database in Poland. Official suicide statistics come from two databases with different data collection mechanisms. These are the General Police Headquarters of Poland (Source: https://statystyka.policja.pl/st/wybrane-statystyki/zamachy-samobojcze, accessed on 26 July 2021) and the Statistics Poland (Source: https://demografia.stat.gov.pl/BazaDemografia/Tables.aspx, accessed on 6 August 2021) [15,16]. The research is based on a secondary analysis of the data obtained from the following:

1. General Police Headquarters of Poland, years 1999–2020. The analysis included suicide attempts, poisoning suicides and suicide attempts, and suicides under the influence of substances affecting the state of consciousness of suicide victims. The General Police Headquarters of Poland (GPHP) is a uniformed and armed force, whose main goal is to serve and protect the people, and to maintain public order and security. Basic responsibilities include protection of people’s health and life, protection of property, protection of public safety and order, organization of “community policing” and crime prevention activities, detection of crimes and misdemeanors, imprisonment of criminals, control of regulations on public life and public spaces, and cooperation with police forces from other countries and international organizations. The data are available to the public. The recorded method of suicide is often imprecise and untrue, which means that such knowledge is limited. The General Police Headquarters of Poland informed that the data concerning a victim may appear in several items.
2. Statistics Poland, years 2002–2020 (data for the years 1999–2001 are not available). The analysis included poisoning deaths according to the International Statistical Classification of Diseases and Related Health Problems ICD-10 (data on suicide attempts are not collected), where the cases of fatal poisonings are classified as intentional and unintentional. Statistics Poland (formerly known in English as the Central Statistical Office [17] (Polish: Główny Urząd Statystyczny, popularly called GUS)) is Poland’s chief government executive agency in charge of collecting and publishing statistics related to the Polish economy, population, and society at the national and local level. The data are available to the public.

The analysis does not include the ICD-10 code X84 “Intentional self-harm by unspecified means”, which could involve poisoning suicide attempts. They are often described as an “external cause of death,” which is imprecise and untrue [4].

In accordance with the statement of the Ethical Committee of the Medical University of Warsaw: “The Committee does not provide opinions on surveys, retrospective studies, or other non-invasive research”; thus, the ethical committee’s consent for the presented research was not required.

### Statistical Analysis

The obtained data were gathered in Microsoft Excel Sheet 2011. Data were analyzed in Inc. STATISTICA, version 13.3. using percentage for categorical variables and mean for continues variables. Chi-squared test was used to analyze categorical variables. *p* values of < 0.05 were considered statistically significant. Intoxication death rates were calculated by dividing the number of deaths due to intoxication by the population of Poland in the respective years 1999–2020, and they were presented per one million of the population. The rate of self-poisoning suicide attempts was calculated analogously.

## 3. Results

### 3.1. Results Based on Data from the General Police Headquarters of Poland

The total of 161,655 cases of suicide attempts in Poland in 1999–2020 resulted in 106,169 deaths (65.7%).

In the analyzed period, the total of 14,660 self-poisoning suicide attempts resulted in 2258 deaths (15.4%).

The total number of suicides of all causes was 106,169 in the 21-year study period. Therefore, the 2258 self-poisoning suicides accounted for 2.1% of the total number of suicides.

The suicide attempt data collected in the period from 1999 to 2020 are shown in Table 1 and distributed by year and intoxication substance.

The results indicated that the majority of suicide attempts in the years 1999–2020 were related to neuroleptic and antipsychotic poisonings (total *n* = 7924). Self-poisoning with other drugs was the cause 4583 suicide attempts. “Designer drugs” or “legal highs” refer to a wide range of products containing novel psychoactive substances (NPS), and this type of self-poisoning accounted for six cases. In the case of 590 suicide attempts, toxic substances causing poisoning were not established.

Table 2 shows categories of substances and poisons that induced death. Neuroleptics and antipsychotics (*n* = 1163) were most often found in the fatalities of self-poisoning suicides in the 21-year observation period. An analysis of death causes revealed that intoxication with gas was the second cause of death (*n* = 457). In the analyzed period, no fatal poisoning with designer drugs was reported, and the toxic substance was not identified in 292 cases of fatal poisonings.

The incidence rate of intoxication deaths showed significant growth in the year 2007 in comparison to the year 2014 (*p* < 0.05). The incidence rate rose significantly in 2017–2020 in comparison to the beginning of 2000s (*p* < 0.05).

Alcohol and other substances are associated with suicide attempts and death by suicide through a number of pathways. The analysis showed that in the analyzed study period alcohol was the most frequently used substance influencing the state of consciousness of the victims of suicide attempts. The lowest number of victims was recorded in 2001 (*n* = 1050) and the highest in 2019 (*n* = 3804). Every year, the state of consciousness of many suicide victims was not established (Table 3).

The statistics for 2017–2020 revealed the emergence of new substances affecting the state of consciousness of suicide victims, and they showed the impact of new psychoactive substances, such as narcotic drugs and drugs (Table 3). It was observed that the number of suicide attempts committed under the influence of these substances has been increasing.

Until 2012, data on suicides were collected in the General Police Headquarters of Poland after the screening was conducted and completed. From 2013, data were entered immediately after the incident, i.e., when it was established that a suicide attempt took place, and the system allows for their modification if it is determined at a later stage of the proceedings that no suicide attempt took place. It is worth mentioning that this change in the data collection strategy was almost immediately reflected in the police statistics, because already in 2013 there was a significant increase in the number of registered suicides, especially suicide attempts (Table 1, Table 2 and Table 3). In the case of the Statistics Poland, data were updated from the death records, and suicide attempts are not reported at all. Such a situation results in significant discrepancies in the reported data, which do not reflect the actual situation and require the development of an optimal form of their registration, enabling reliable data.

Cases of death by suicide depending on the state of consciousness have been listed in the statistics since 2017 (Table 4). Since 2017, there has been a steady decline in the number of suicides under the influence of alcohol. In 2017, alcohol was found in 602 suicide victims, whereas in the year 2020 it was found 521 suicides.

### 3.2. Results Based on Data Obtained from the Statistics Poland

Our study indicates that, in the analyzed observation period, alcohol was frequently responsible for intentional (suicidal) (Table 5) and unintentional deaths as the cause of poisonings (Table 6). Antiepileptic, sedative-hypnotic, anti-parkinsons, and psychotropic drugs, not elsewhere classified, as well as other unspecified drugs, medicaments, and biological substances were the second cause of death as a result of intentional self-poisoning (Table 5). Poisoning by and exposure to other unspecified drugs, medicaments, and biological substances, as well as poisoning by and exposure to other gases and vapors, were the second cause of deaths as a result of undetermined intent (Table 6).

### 3.3. Other Results

**Figure 1:**
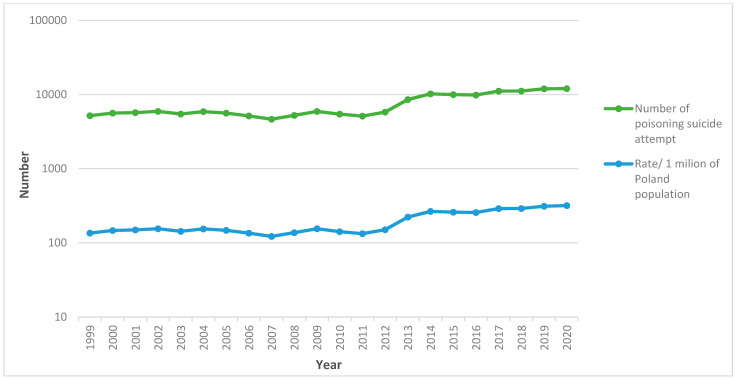
The numbers of suicide attempt and population rates caused by intoxication per 1 million of the population, Poland, 1999–2020 (source: own study).

Figure 1 shows the annual number and rate of poisoning suicide attempts per 1 million of the population in Poland. The rate of total poisoning suicide attempts was determined as the lowest in 2007 (122.21/1 million of the population) and the highest in 2020 (318.36/1 million of the population).

**Figure 2:**
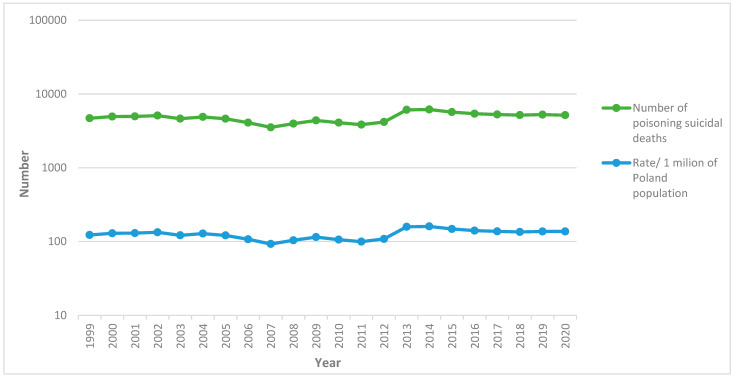
The numbers of suicide deaths and population rates caused by intoxication per 1 million of the population, Poland, 1999–2020 (source: own study). Note: The population of Poland was quite constant over the period of the study. Poland has a population of nearly 38 million people [18].

Figure 2 shows the incidence rate of suicide deaths caused by intoxication per 1 million of the population in Poland in the 21-year study period. The rate of total poisoning suicide deaths was determined as the lowest in 2007 (92.61/1 million of the population) and the highest in 2014 (160.22/1 million of the population).

## 4. Discussion

Relative frequencies of various methods used for self-harm have varied over time, and they do vary by setting, population type, and country [19]. Within most population groups presenting to medical services, 75%–90% cases of self-harm involve poisoning by over the counter or prescription medications, 10% are related to self-cutting, and less than 5% involve violent methods (e.g., hanging, shooting, jumping from heights), ingestion of toxic chemicals (e.g., bleach, antifreeze) or severe self-injury (e.g., stabbing, burning) [20].

It is difficult to obtain reliable information on the morbidity and mortality resulting from poisoning, even in countries with comparatively advanced population health data collection systems. Despite difficulties in the interpretation of available data, certain general observations can be made on the epidemiology of poisoning. Childhood poisoning is usually accidental and tends to be associated with low morbidity and mortality. In Western Europe and North America, it most often involves the use of household products and pharmaceuticals, whereas in developing countries, pesticides and household products are most commonly used. In adults, self-poisoning is usually deliberate (suicide or parasuicide), and it has higher morbidity and mortality than other methods [21].

Self-poisoning suicide is very difficult to recognize. It is not only because of the necessity to determine the suicide while excluding the possibility of a murder or an accident, but also in specific cases it is necessary to consider the possibility of death from poisoning, and not from natural causes. In most situations, the poisons used for suicide do not cause any characteristic pathological changes or specific clinical symptoms. Even if it has already been established that the cause of death was the use of a toxic substance, determination of a suicide poses serious difficulties, because in the case of criminal poisoning, the perpetrator tries to make it look like a suicide or accidental poisoning. It is important to determine what chemical agent the suicide was caused by. Time is a factor that significantly influences the effectiveness of toxicological testing [22].

Statistics on suicides in Poland are not clear. Data on the number of suicides per year are provided by the General Police Headquarters of Poland (the GPHP) and the Statistics Poland (GUS). The GPHP base their analysis on the KSIP-10 report on suicide attempt/behavior. It is not necessary to introduce the social security number of a victim there. GUS creates their reports based on death cards. Differences between the reporting systems lead to the discrepancies in the numbers of suicides, depending on the source of information.

Until 2013, the statistics of the GPHP always showed the number of suicides, which was about 2000 less than the number reported by the GUS. In the year 2013, GPHP changed the method of collecting and generating statistical data on suicide attempts. Previously, the data were entered into the system after an investigation and termination of procedures. Presently, if the circumstances indicate that it came to a suicide attempt, data are implemented directly after the accident. The system allows for the modification of data when it turns out that the accident was not an attempt. In the year 2013, the media reported a sharp increase in the number of suicides in Poland (according to the GPHP, the number of suicides increased by about 2000). This fact was not caused by the actual growth of the suicide rate in Poland but because of the implementation of changes to the GPHP reporting system. In 2017, the KSIP-10 form (registration of a suicide) was changed. It received the wording “KSIP-10 report on suicide attempt/behavior” and the scope of the collected data was extended. It is necessary to analyze suicide statistics in Poland very carefully [23].

Self-poisoning is one of the most common methods used to attempt suicide in Poland [24]. Nearly 70% of suicide victims consume a toxic substance prior to death, and the primary method used in one in five suicides in the United States in 2006–2008 was overdose [25]. According to Cavanagh and Smith [9], self-poisoning is a common method, accounting for over half of all female suicides. Fatal self-poisoning most frequently involves pesticides, analgesics, and antidepressants. Overdoses of illicit substances, such as heroin, are also common, but the intention to die may be difficult to determine.

According to the GPHP, intoxication with neuroleptics or antipsychotics has been the most common method for suicide attempts in Poland in the last 21 years. This group of pharmaceuticals was also responsible for the majority of suicide deaths. Intoxication with other medications was the second method for suicide attempts and third cause of suicide deaths. Intoxication with gas was the second cause of suicide deaths and the third method for suicide attempts. In a study by Methling et al. [26], in which all cases (*n* = 477) with positive toxicology for antidepressants and antipsychotics in blood or organ tissue were included, the prevalence of substances detected in non-suicide cases (*n* = 212; male *n* = 177, 55.2%; female *n* = 95, 52.5%) and suicide cases (*n* = 235; male *n* = 149, 63.4%; female *n* = 86, 36.6%) was examined. Tricyclic antidepressants (48.1%) were most frequent in suicides, followed by atypical neuroleptics (37.0%), selective serotonin reuptake inhibitors (28.1%), typical neuroleptics (17.4%), tetracyclic antidepressants (16.2%), and other substances (8.9%). Alcohol was detected in 37.2% of suicides. An analysis by Hołyst [22] based on the suicide statistics in Poland in 1994–2000 showed that every year about 170 people use sleeping pills, 60% of suicide victims use poison, and 40% use gas to commit suicide. Substances that are the most commonly used for suicide are currently sleeping pills. In the past, almost exclusively barbiturates (luminal, veronal, fanodorm) were used, along with glutethimide (glimide) and followed by tardyl. Other psychotropic drugs are shown elsewhere in the statistics of suicide poisoning. Barbiturates show strong toxic synergism with ethyl alcohol. Poisoning with barbiturates is accompanied by symptoms such as headache, visual disturbances, motor agitation, and in the case of acute poisoning, coma, loss of consciousness, lower body temperature, weak pulse, and low blood pressure. Death occurs as a result of the paralysis of the respiratory system. Anatomical changes are poorly visible during the autopsy. A typical representative of barbiturates is the popular hypnotic luminal. Pharmacological agents used by suicides are not limited to barbiturates. In the case of other agents, a delay in the post-mortem examination can make it impossible to determine the role of aspirin in causing death, as aspirin does not induce any characteristic anatomical changes and disappears in the body over time. Another pharmaceutical that is used for self-poisoning is strychnine; however, it is rare. Thallium and cyanides are also popular with suicide victims [22]. Chemical compounds are also applied, with the most popular of them being organophosphorus compounds, cyanides, nitrites, and even potassium permanganate. A suicide victim does not feel embarrassed by the amount of poison taken. They take it in large amounts, sometimes even several dozen or more tablets of drugs. Several types of poison are often taken simultaneously. Using one substance is more frequently associated with a murder [22].

A large group of suicide attempters in Poland in 1999–2020 were under the influence of alcohol, but since 2017 there has been a steady decline in the number of suicide deaths under the influence of alcohol. Being the cause of self-poisoning, alcohol was frequently responsible for intentional (suicidal) and unintentional deaths. It is especially difficult to distinguish suicide from accidental poisoning. It is quite common that a drunk person drinks various poisonous liquids, in the belief that these are alcoholic drinks. This happens when poisonous liquids are stored in bottles with labels of vodkas, wines, or other alcoholic drinks. Additionally, a wrong combination of drugs, especially prescribed by several doctors, can cause a strong toxic effect that is dangerous to health and life [22].

In the present study, suicide attempts were often caused by the intake of sedatives and anxiolytics. Self-poisoning with this group of medicines is common in urban areas throughout the tropics, in Western Europe, North America, the United Kingdom, Finland, and Tehran, Iran [27]. With regard to anxiolytic and sedative medicines, benzodiazepines were the most frequently used [21]. A review of hospital reports related to self-harm in England showed that overdose was the cause of 79% of cases, self-laceration was observed in 11% of patients, and a combination of the two in 5% of self-harm victims. Other methods included self-poisoning with toxic substances (e.g., bleach), attempted hanging, jumping from height, and carbon monoxide poisoning. With regard to the self-poisoning episodes, paracetamol was used in 50% of cases, antidepressants in 20%, aspirin in 10%, and other drugs in the remaining cases [28]. An Australian study by Cairns et al. [29] showed that children and adolescents (5–19 years old) most commonly used the following substances for self-poisonings: paracetamol, ibuprofen, fluoxetine, ethanol, quetiapine, paracetamol/opioid combinations, sertraline, and escitalopram. There was an increase in psychotropic dispensing, the use of selective serotonin reuptake inhibitors (SSRIs) and antipsychotics. Conversely, dispensing of benzodiazepines to these age groups decreased.

Regarding the data presented in this study, no fatal self-poisoning with designer drugs has been reported. However, the national average of poisoning cases with designer drugs in Poland in 2015 was 18.92/100,000 inhabitants [30].

Carbon monoxide poisoning is a relatively common method of suicide. Suicidal poisoning refers to intoxication with carbon monoxide contained in the light gas. Suicide cannot be ruled out when a dead body is found in a garage and the CO is released in car exhaust fumes [22].

## 5. Conclusions

Over the last 21 years, 106,169 suicides have been committed in Poland, 2258 of which were self-poisoning suicides (accounting for about 2.1% of all suicide deaths). The analysis of statistical data shows that it is important to carefully interpret data on suicides in Poland, considering both sources of information: GPHP and GUS. Obligatory and reliable reporting of all suicides and suicide attempts to a uniform database should be done to improve surveillance. Neuroleptics and antipsychotics were the most frequently used drugs for self-poisoning. Alcohol intoxication was also very common among suicide attempters. The highest rate of self-poisoning suicide deaths was recorded in 2014, while the highest rate of suicide attempts related to self-poisoning was recorded in 2020. These results indicate an increased interest in poisoning for suicidal purposes, which is why appropriate preventive measures should be introduced in order to limit the occurrence of this phenomenon. Basic activities should be aimed at the entire population, and they should involve strengthening personal and social competences to effectively cope with stress and developing knowledge of what to do in the event of a mental crisis. Additionally, it is important to provide those suffering from a mental crisis with access to specialist care and information on available forms of support. An effective activity in the field of suicide prevention is restriction of access to certain suicide methods. Hopes are pinned on the National Suicide Prevention Program, which is to be implemented in Poland for the first time under the National Health Program 2021–2025.

## 6. Limitations

This study has some limitations, including the following: (1) information on mixed poisoning was not found; (2) considering the changes in the registration procedures, not all data are available for specific periods of time; (3) the study lacks information about suicide attempts and suicide victims with reference to the state of consciousness as affected by unspecified/not established agents and methods of suicide.

Despite these limitations, the study provides basic information about suicide attempts, suicide in general, and self-poisoning suicides, and because access to nationwide data was an advantage to data analysis and completeness, the study allows for a comparison with international data.

## Figures and Tables

**Table 1:** Substances causing suicide attempts in 1999–2020 in Poland (source: own study based on data by GPHP).

Year	Total Number of Suicide Attempts	Intoxication of Gases	Intoxication of Chemicals	Intoxication Neuroleptics and Antipsychotics	Intoxication of Other Medications	Intoxication of Substance Abuse	Intoxication of Designer Drugs	Intoxication of Unspecified Substances
1999	5182	33	-	153	-	-	-	28
2000	5621	33	-	169	-	-	-	48
2001	5712	32	-	175	-	-	-	41
2002	5928	29	-	211	-	-	-	30
2003	5467	33	-	211	-	-	-	21
2004	5893	43	-	220	-	-	-	23
2005	5625	43	-	188	-	-	-	22
2006	5152	31	-	187	-	-	-	26
2007	4658	29	-	198	-	-	-	23
2008	5237	24	-	190	-	-	-	28
2009	5913	41	-	199	-	-	-	30
2010	5456	38	-	214	-	-	-	26
2011	5124	39	-	192	-	-	-	20
2012	5791	33	-	193	-	-	-	30
2013	8575	47	-	314	-	-	-	41
2014	10,207	67	-	474	-	-	-	52
2015	9973	85	-	520	-	-	-	52
2016	9861	73	-	572	-	-	-	49
2017	11,139	92	62	674	959	21	3	-
2018	11,167	104	68	774	1061	17	3	-
2019	11,961	114	89	894	1289	19	0	-
2020	12,013	102	106	1002	1274	10	0	-
TOTAL	161,655	1165	325	7924	4583	67	6	590

**Table 2:** Suicidal deaths by substance in 1999–2020 in Poland (source: own study based on data by GPHP).

Year	Total Number of Suicidal Deaths	Intoxication of Gases	Intoxication of Chemicals	Intoxication Neuroleptics and Antipsychotics	Intoxication of Other Medications	Intoxication of Substance Abuse	Intoxication of Designer Drugs	Intoxication of Unspecified Substances
1999	4695	25	-	56	-	-	-	19
2000	4947	19	-	66	-	-	-	34
2001	4971	17	-	67	-	-	-	16
2002	5100	19	-	74	-	-	-	23
2003	4634	19	-	60	-	-	-	17
2004	4893	20	-	70	-	-	-	16
2005	4621	15	-	61	-	-	-	14
2006	4090	17	-	39	-	-	-	17
2007	3530	10	-	37	-	-	-	12
2008	3964	7	-	43	-	-	-	16
2009	4384	11	-	33	-	-	-	15
2010	4087	17	-	38	-	-	-	11
2011	3839	25	-	41	-	-	-	9
2012	4177	13	-	37	-	-	-	13
2013	6101	15	-	59	-	-	-	14
2014	6165	18	-	69	-	-	-	14
2015	5688	37	-	45	-	-	-	19
2016	5405	21	-	66	-	-	-	13
2017	5276	29	17	44	58	4	0	-
2018	5182	38	12	48	61	3	0	-
2019	5255	41	24	50	63	4	0	-
2020	5165	24	32	60	67	1	0	-
TOTAL	106,169	457	85	1163	249	12	0	292

**Table 3:** Suicide attempts depending on the state of consciousness (source: own study based on data by GPHP).

Year	Sober *	Under the Influence of Alcohol *	Under the Influence of Psychotropic Substance (Drugs) *	Under the Influence of Medications *	Under the Influence of Substitute Agents/Substances (Designer Drugs) *	Not Specified/Agent Not Established *
1999	866	1056	54	-	28	3199
2000	855	1134	80	-	35	3543
2001	851	1050	49	-	26	3754
2002	815	1069	80	-	41	3953
2003	686	1059	44	-	30	3667
2004	792	1189	58	-	31	3849
2005	737	1244	59	-	29	3575
2006	669	1120	54	-	37	3291
2007	644	1069	41	-	30	2897
2008	605	1255	53	-	41	3310
2009	668	1453	44	-	44	3733
2010	592	1341	32	-	47	3472
2011	500	1258	27	-	31	3334
2012	618	1438	45	-	47	3676
2013	683	1857	62	-	66	5974
2014	901	2734	109	-	106	6432
2015	867	2841	133	-	118	6093
2016	987	2899	113	-	98	5815
2017	1306	3635	79	734	31	5575
2018	1342	3634	92	809	21	5526
2019	1571	3804	98	929	22	5777
2020	1700	3756	102	1057	15	5675

Legend: * substances concerning a suicide attempt may appear in several items; -data not collected.

**Table 4:** Suicide depending on the state of consciousness (source: own study based on data by GPHP).

Year	Sober *	Under the Influence of Alcohol *	Under the Influence of Psychotropic Substance (Drugs) *	Under the Influence of Medications *	Under the Influence of Substitute Agents/Substances (Designer Drugs) *	Not Specified/Agent Not Established *
1999	-	-	-	-	-	-
2000	-	-	-	-	-	-
2001	-	-	-	-	-	-
2002	-	-	-	-	-	-
2003	-	-	-	-	-	-
2004	-	-	-	-	-	-
2005	-	-	-	-	-	-
2006	-	-	-	-	-	-
2007	-	-	-	-	-	-
2008	-	-	-	-	-	-
2009	-	-	-	-	-	-
2010	-	-	-	-	-	-
2011	-	-	-	-	-	-
2012	-	-	-	-	-	-
2013	-	-	-	-	-	-
2014	-	-	-	-	-	-
2015	-	-	-	-	-	-
2016	-	-	-	-	-	-
2017	341	602	13	69	3	4276
2018	323	570	12	54	0	4245
2019	324	526	16	59	2	4350
2020	360	521	11	64	1	4229

Legend: * substances concerning a suicide may appear in several items; -data not collected.

**Table 5:** Number of deaths in the category: intentional self-harm (suicide) X60–X69 according to ICD-10, in 1999–2020 in Poland (source: own study based on data by GUS).

Year	ICD-10 Classification										
	X60	X61	X62	X63	X64	X65	X66	X67	X68	X69	TOTAL
2002	1	69	8	6	75	10	0	16	1	10	196
2003	2	76	4	1	66	7	4	17	4	17	198
2004	6	70	7	2	54	2	1	21	4	14	181
2005	2	57	4	2	66	8	3	23	3	15	183
2006	4	56	4	4	60	6	3	24	3	20	184
2007	2	43	3	3	47	3	0	11	6	7	125
2008	1	55	3	4	46	10	4	11	5	16	155
2009	5	46	12	2	41	133	7	43	2	18	308
2010	2	45	6	2	58	308	2	21	1	17	462
2011	5	50	8	3	34	311	4	26	2	11	454
2012	1	31	8	3	44	487	0	27	1	7	609
2013	0	24	9	1	53	617	2	10	0	3	719
2014	3	22	5	3	41	564	0	8	1	3	650
2015	2	33	0	10	44	368	1	19	3	6	486
2016	3	24	7	3	36	150	1	13	3	4	244
2017	3	29	9	0	35	117	2	9	2	4	210
2018	3	34	10	4	33	137	1	15	1	6	244
2019	3	37	9	0	48	121	2	13	2	0	235
2020	3	32	13	1	38	230	1	8	1	13	340
TOTAL	51	833	129	54	919	3589	38	335	45	191	6183

Legend: X60—Intentional self-poisoning by and exposure to nonopioid analgesics, antipyretics, and antirheumatics; X61—Intentional self-poisoning by and exposure to antiepileptic, sedative-hypnotic, anti-parkinson, and psychotropic drugs, not elsewhere classified; X62—Intentional self-poisoning by and exposure to narcotics and psychodysleptics hallucinogens), not elsewhere classified; X63—Intentional self-poisoning by and exposure to other drugs acting on the autonomic nervous system; X64—Intentional self-poisoning by and exposure to other and unspecified drugs, medicaments, and biological substances; X65—Intentional self-poisoning by exposure to alcohol; X66—Intentional self-poisoning by and exposure to organic solvents and halogenated hydrocarbons and their vapors; X67—Intentional self-poisoning by and exposure to other gases and vapors; X68—Intentional self-poisoning by and exposure to pesticides; X69—Intentional self-poisoning by and exposure to other and unspecified chemicals and noxious substances.

**Table 6:** Number of deaths in the category: undetermined intent (suicide) X60–X69 according to ICD-10, in 1999–2020 in Poland (source: own study based on data by GUS).

Year	ICD-10 Classification										
	Y10	Y11	Y12	Y13	Y14	Y15	Y16	Y17	Y18	Y19	TOTAL
2002	2	27	34	1	95	134	7	45	8	48	401
2003	4	32	30	2	75	121	15	37	9	51	376
2004	1	24	32	2	86	102	11	33	1	45	337
2005	1	20	28	1	83	176	18	53	3	37	420
2006	2	21	23	2	80	232	9	51	3	58	481
2007	4	23	37	3	67	522	9	70	6	52	793
2008	5	22	62	2	76	615	8	101	6	29	926
2009	1	25	61	3	70	388	9	55	0	27	639
2010	2	20	82	0	64	415	16	119	3	31	752
2011	1	27	90	2	71	538	4	91	2	33	859
2012	1	11	80	0	58	427	6	107	2	20	712
2013	2	9	77	1	46	407	4	93	3	14	656
2014	3	15	102	3	57	379	5	51	0	10	625
2015	5	16	96	1	45	386	2	56	0	22	629
2016	2	22	67	1	37	275	2	65	1	14	486
2017	4	12	26	0	30	162	6	63	1	20	324
2018	1	11	27	1	23	141	1	73	2	37	317
2019	3	14	36	2	34	102	2	42	2	16	253
2020	1	12	17	0	30	85	2	31	1	15	194
TOTAL	45	363	1007	27	1127	5607	136	1236	53	579	10180

Legend: Y10—Poisoning by and exposure to nonopioid analgesics, antipyretics, and antirheumatics, undetermined intent; Y11—Poisoning by and exposure to antiepileptic, sedative-hypnotic, anti-parkinson, and psychotropic drugs, not elsewhere classified, undetermined intent; Y12—Poisoning by and exposure to narcotics and psychodysleptics (hallucinogens), not elsewhere classified, undetermined intent; Y13—Poisoning by and exposure to other drugs acting on the autonomic nervous system, undetermined intent; Y14—Poisoning by and exposure to other and unspecified drugs, medicaments, and biological substances, undetermined intent; Y15—Poisoning by and exposure to alcohol, undetermined intent; Y16—Poisoning by and exposure to organic solvents and halogenated hydrocarbons and their vapors, undetermined intent; Y17—Poisoning by and exposure to other gases and vapors, undetermined intent; Y18—Poisoning by and exposure to pesticides, undetermined intent; Y19—Poisoning by and exposure to other and unspecified chemicals and noxious substances, undetermined intent.

## Data Availability

Data of General Police Headquarters of Poland: https://statystyka.policja.pl/st/wybrane-statystyki/zamachy-samobojcze (accessed on 27 December 2021). Data of Statistics Poland: https://demografia.stat.gov.pl/BazaDemografia/Tables.aspx (accessed on 27 December 2021).

## Abbreviations

GPHP	General Police Headquarters of Poland
GUS	Statistics Poland
NPS	novel psychoactive substances
OTC	over the counter
WHO	World Health Organization
